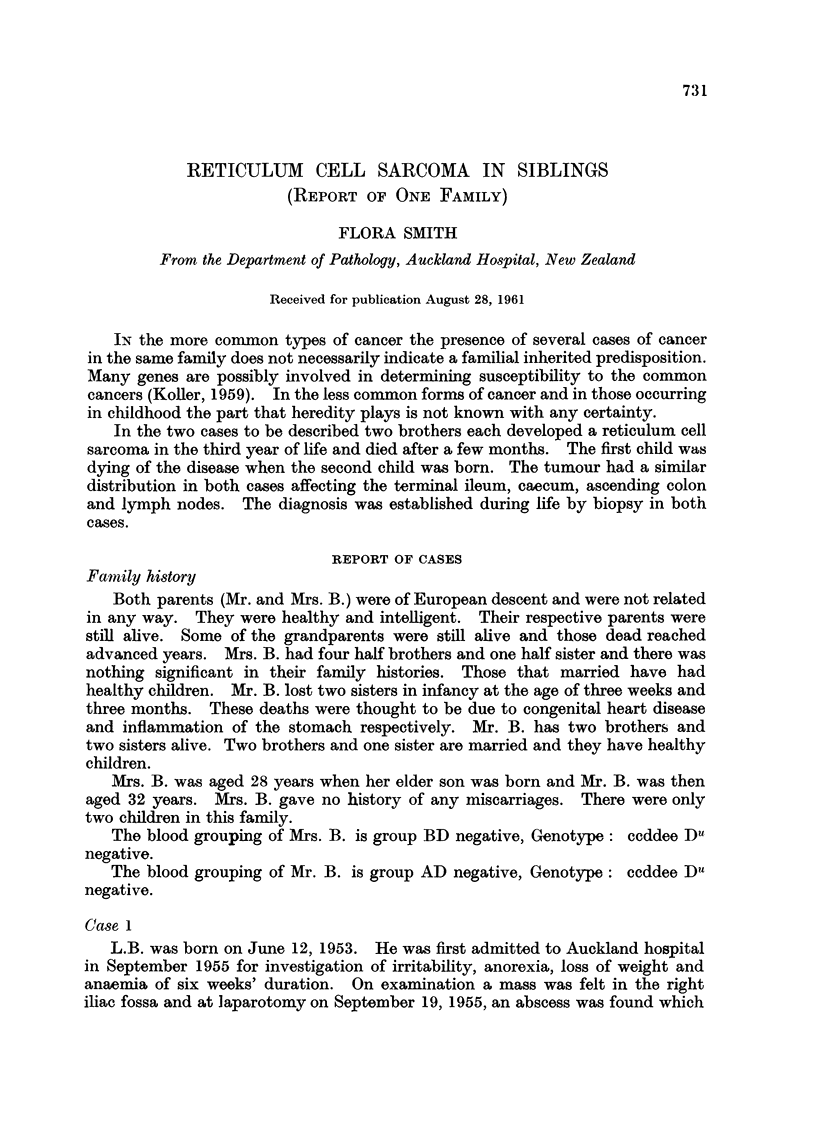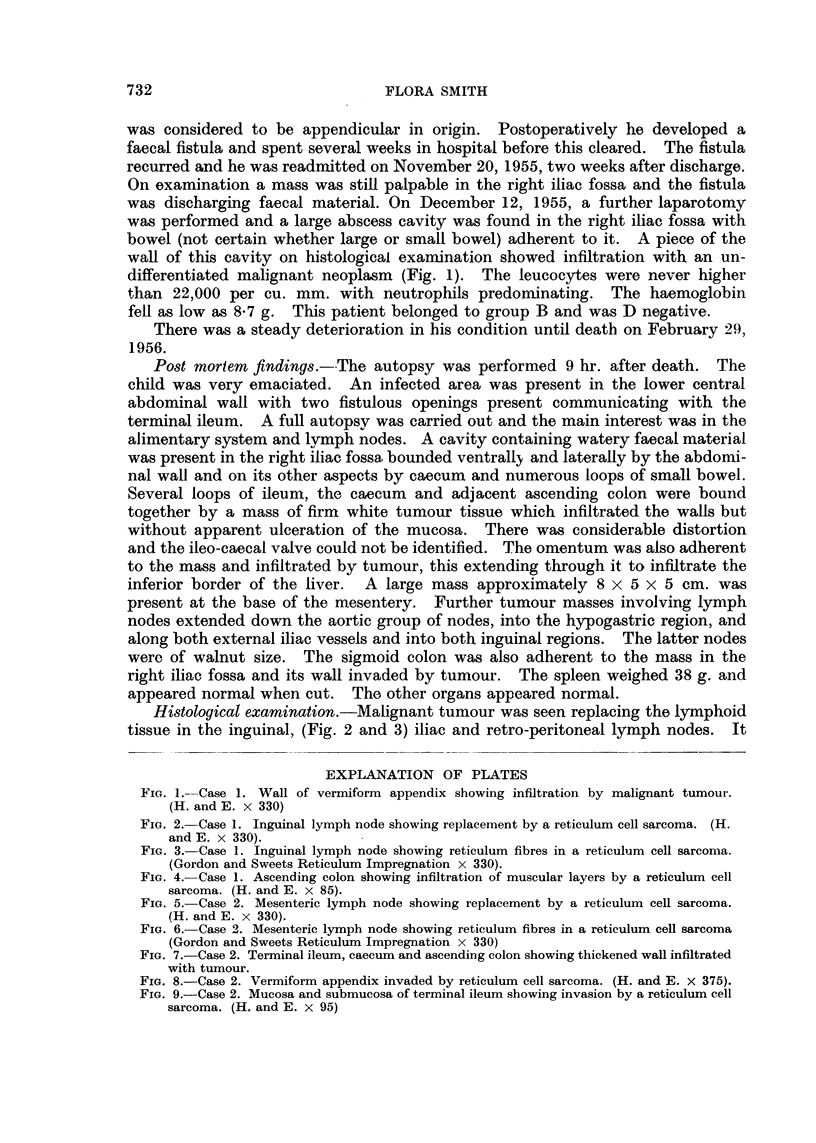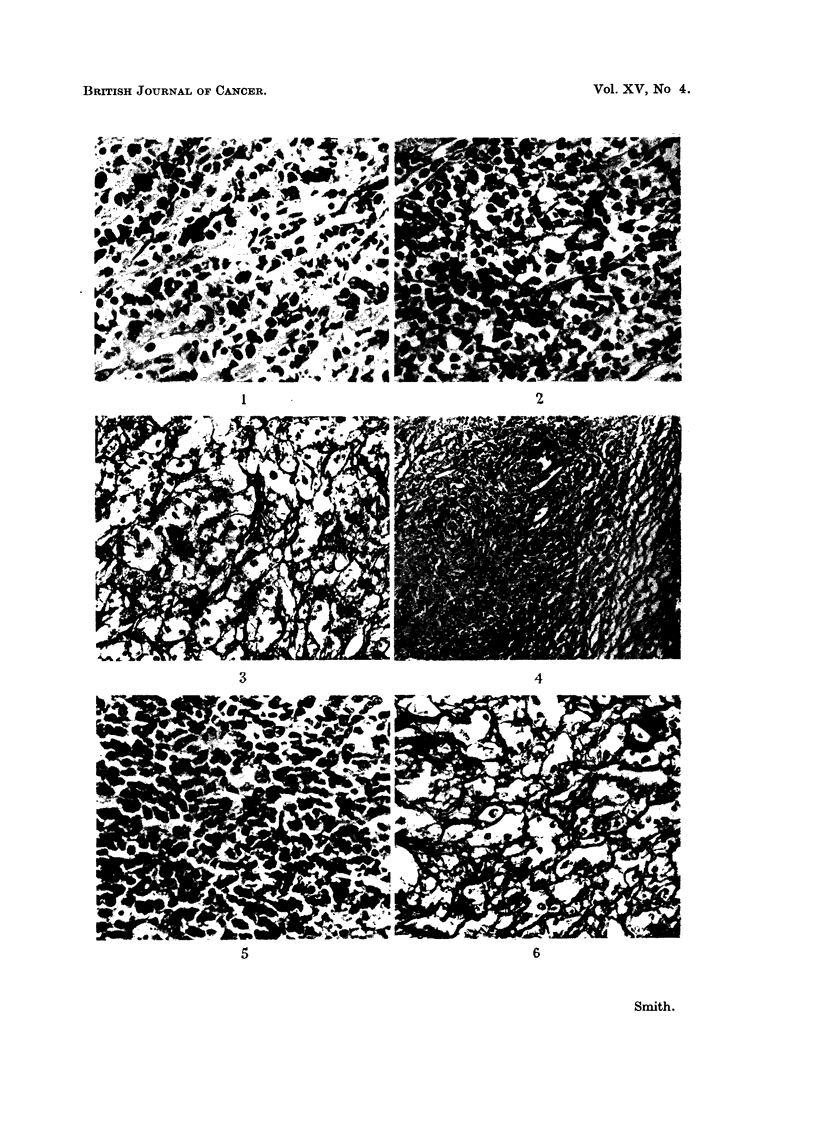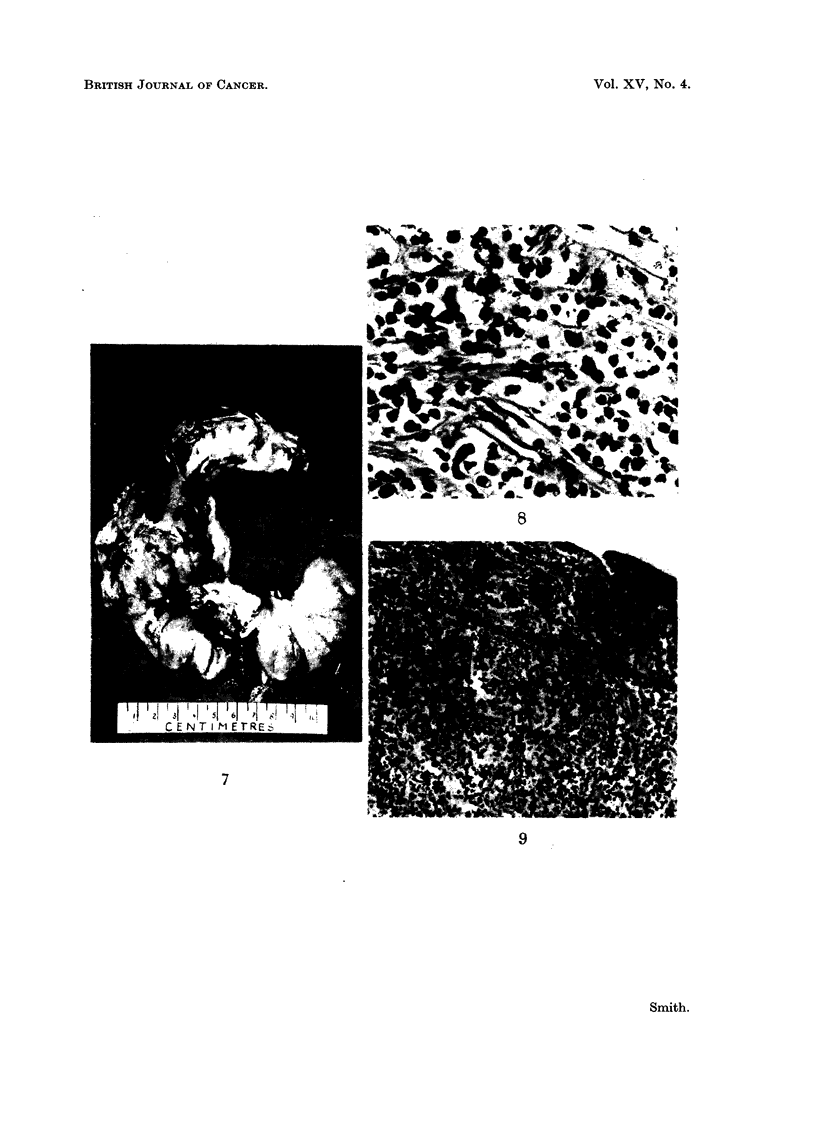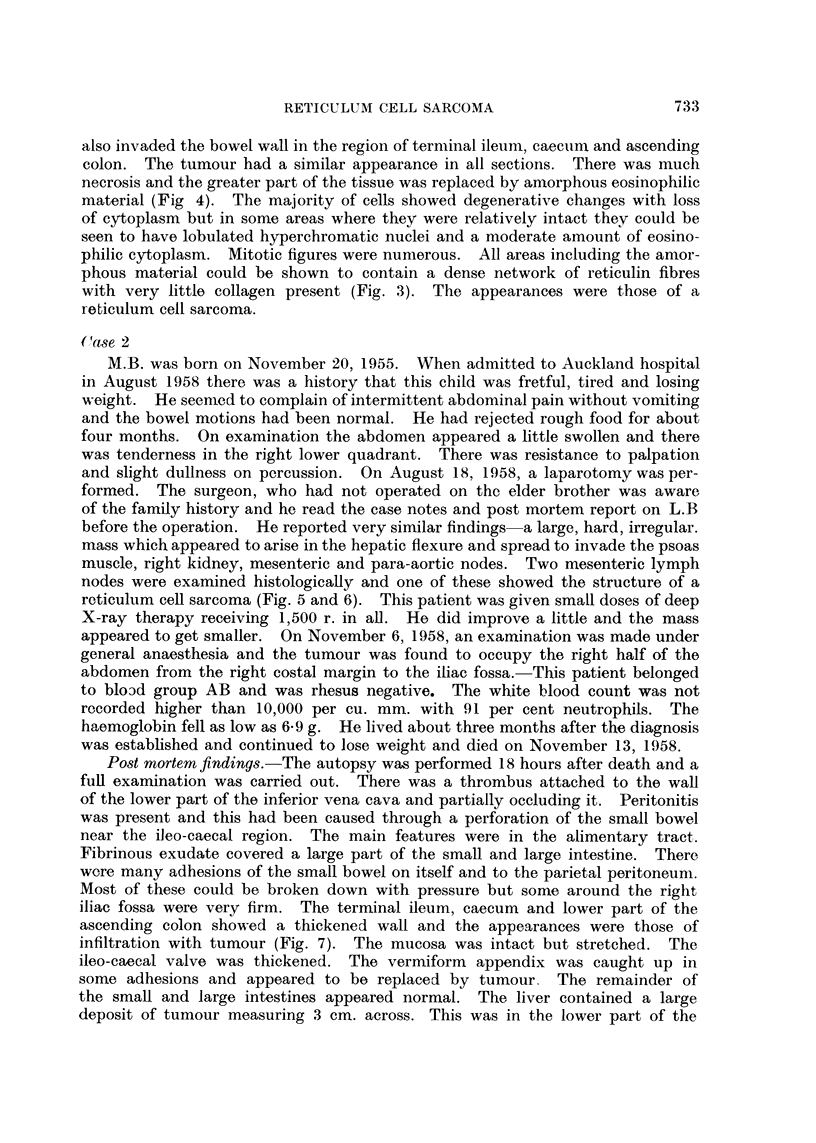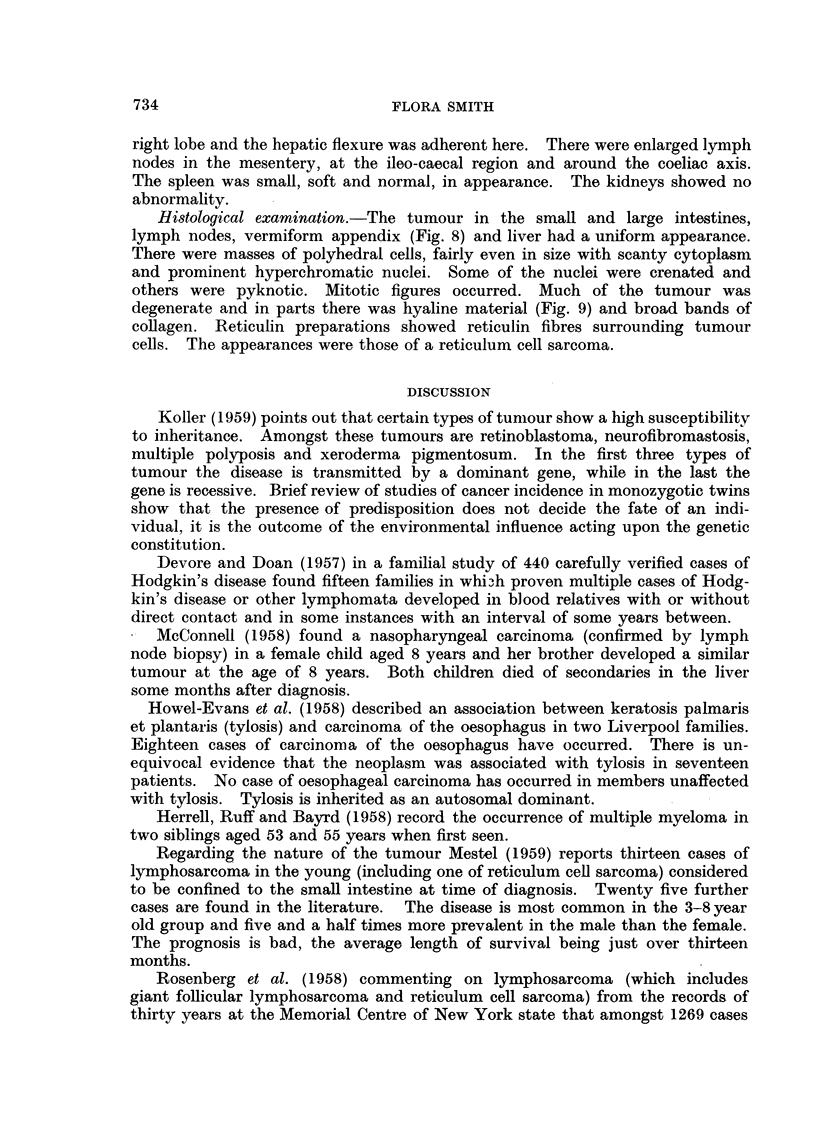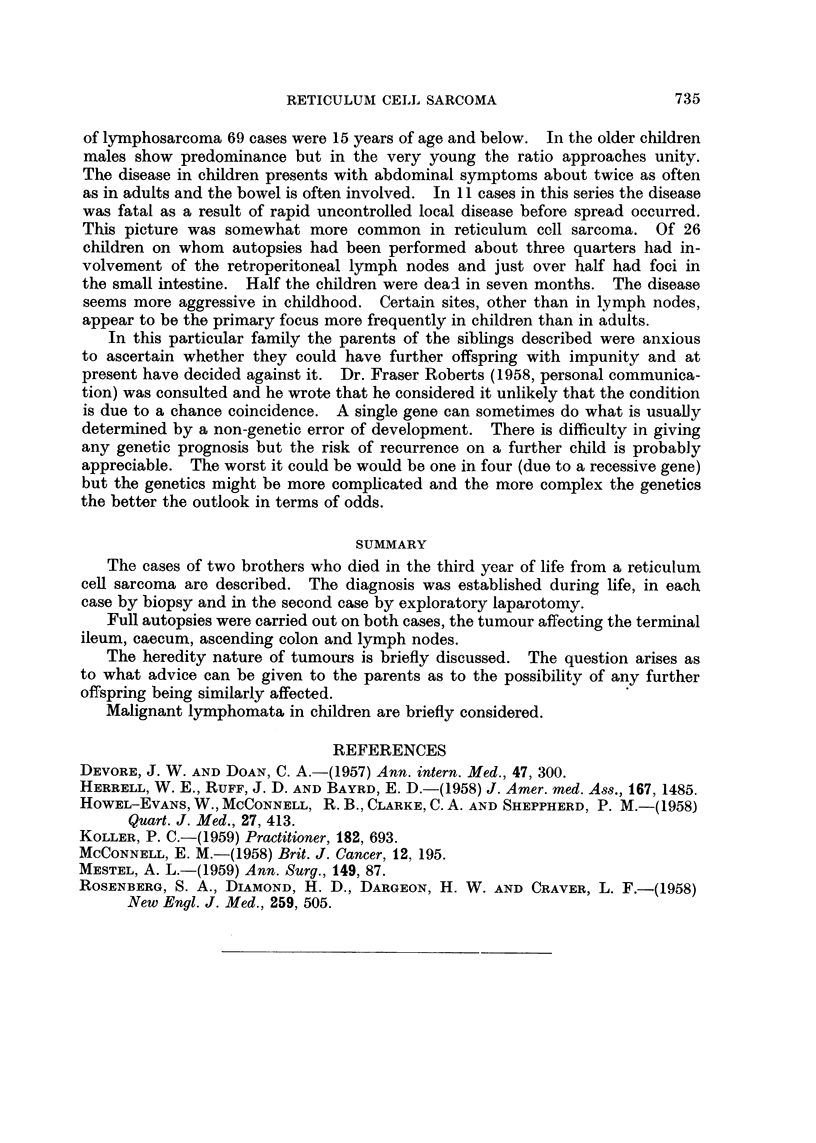# Reticulum Cell Sarcoma in Siblings (Report of One Family)

**DOI:** 10.1038/bjc.1961.83

**Published:** 1961-12

**Authors:** Flora Smith

## Abstract

**Images:**


### RETICULUM CELL SARCOMA IN SIBLINGS
### (Report of One Family)

FLORA SMITH

*From the Department of Pathology, Auckland Hospital, New Zealand*

Received for publication August 28, 1961

In the more common types of cancer the presence of several cases of cancer in the same family does not necessarily indicate a familial inherited predisposition. Many genes are possibly involved in determining susceptibility to the common cancers (Koller, 1959). In the less common forms of cancer and in those occurring in childhood the part that heredity plays is not known with any certainty.

In the two cases to be described two brothers each developed a reticulum cell sarcoma in the third year of life and died after a few months. The first child was dying of the disease when the second child was born. The tumour had a similar distribution in both cases affecting the terminal ileum, caecum, ascending colon and lymph nodes. The diagnosis was established during life by biopsy in both cases.

### REPORT OF CASES

*Family history*

Both parents (Mr. and Mrs. B.) were of European descent and were not related in any way. They were healthy and intelligent. Their respective parents were still alive. Some of the grandparents were still alive and those dead reached advanced years. Mrs. B. had four half brothers and one half sister and there was nothing significant in their family histories. Those that married have had healthy children. Mr. B. lost two sisters in infancy at the age of three weeks and three months. These deaths were thought to be due to congenital heart disease and inflammation of the stomach respectively. Mr. B. has two brothers and two sisters alive. Two brothers and one sister are married and they have healthy children.

Mrs. B. was aged 28 years when her elder son was born and Mr. B. was then aged 32 years. Mrs. B. gave no history of any miscarriages. There were only two children in this family.

The blood grouping of Mrs. B. is group BD negative, Genotype : ccddee $D^u$ negative.

The blood grouping of Mr. B. is group AD negative, Genotype : ccddee $D^u$ negative.

*Case* 1

L.B. was born on June 12, 1953. He was first admitted to Auckland hospital in September 1955 for investigation of irritability, anorexia, loss of weight and anaemia of six weeks' duration. On examination a mass was felt in the right iliac fossa and at laparotomy on September 19, 1955, an abscess was found which was considered to be appendicular in origin. Postoperatively he developed a faecal fistula and spent several weeks in hospital before this cleared. The fistula recurred and he was readmitted on November 20, 1955, two weeks after discharge. On examination a mass was still palpable in the right iliac fossa and the fistula was discharging faecal material. On December 12, 1955, a further laparotomy was performed and a large abscess cavity was found in the right iliac fossa with bowel (not certain whether large or small bowel) adherent to it. A piece of the wall of this cavity on histological examination showed infiltration with an undifferentiated malignant neoplasm (Fig. 1). The leucocytes were never higher than 22,000 per cu. mm. with neutrophils predominating. The haemoglobin fell as low as 8·7 g. This patient belonged to group B and was D negative.

There was a steady deterioration in his condition until death on February 29, 1956.

*Post mortem findings.*—The autopsy was performed 9 hr. after death. The child was very emaciated. An infected area was present in the lower central abdominal wall with two fistulous openings present communicating with the terminal ileum. A full autopsy was carried out and the main interest was in the alimentary system and lymph nodes. A cavity containing watery faecal material was present in the right iliac fossa bounded ventrally and laterally by the abdominal wall and on its other aspects by caecum and numerous loops of small bowel. Several loops of ileum, the caecum and adjacent ascending colon were bound together by a mass of firm white tumour tissue which infiltrated the walls but without apparent ulceration of the mucosa. There was considerable distortion and the ileo-caecal valve could not be identified. The omentum was also adherent to the mass and infiltrated by tumour, this extending through it to infiltrate the inferior border of the liver. A large mass approximately $8 \times 5 \times 5$ cm. was present at the base of the mesentery. Further tumour masses involving lymph nodes extended down the aortic group of nodes, into the hypogastric region, and along both external iliac vessels and into both inguinal regions. The latter nodes were of walnut size. The sigmoid colon was also adherent to the mass in the right iliac fossa and its wall invaded by tumour. The spleen weighed 38 g. and appeared normal when cut. The other organs appeared normal.

*Histological examination.*—Malignant tumour was seen replacing the lymphoid tissue in the inguinal, (Fig. 2 and 3) iliac and retro-peritoneal lymph nodes. It

---

EXPLANATION OF PLATES

FIG. 1.—Case 1. Wall of vermiform appendix showing infiltration by malignant tumour. (H. and E. × 330)

FIG. 2.—Case 1. Inguinal lymph node showing replacement by a reticulum cell sarcoma. (H. and E. × 330).

FIG. 3.—Case 1. Inguinal lymph node showing reticulum fibres in a reticulum cell sarcoma. (Gordon and Sweets Reticulum Impregnation × 330).

FIG. 4.—Case 1. Ascending colon showing infiltration of muscular layers by a reticulum cell sarcoma. (H. and E. × 85).

FIG. 5.—Case 2. Mesenteric lymph node showing replacement by a reticulum cell sarcoma. (H. and E. × 330).

FIG. 6.—Case 2. Mesenteric lymph node showing reticulum fibres in a reticulum cell sarcoma (Gordon and Sweets Reticulum Impregnation × 330)

FIG. 7.—Case 2. Terminal ileum, caecum and ascending colon showing thickened wall infiltrated with tumour.

FIG. 8.—Case 2. Vermiform appendix invaded by reticulum cell sarcoma. (H. and E. × 375).

FIG. 9.—Case 2. Mucosa and submucosa of terminal ileum showing invasion by a reticulum cell sarcoma. (H. and E. × 95)

1

2

3

4

5

6

Smith.

7

8

9

Smith.

also invaded the bowel wall in the region of terminal ileum, caecum and ascending colon. The tumour had a similar appearance in all sections. There was much necrosis and the greater part of the tissue was replaced by amorphous eosinophilic material (Fig 4). The majority of cells showed degenerative changes with loss of cytoplasm but in some areas where they were relatively intact they could be seen to have lobulated hyperchromatic nuclei and a moderate amount of eosinophilic cytoplasm. Mitotic figures were numerous. All areas including the amorphous material could be shown to contain a dense network of reticulin fibres with very little collagen present (Fig. 3). The appearances were those of a reticulum cell sarcoma.

*Case 2*

M.B. was born on November 20, 1955. When admitted to Auckland hospital in August 1958 there was a history that this child was fretful, tired and losing weight. He seemed to complain of intermittent abdominal pain without vomiting and the bowel motions had been normal. He had rejected rough food for about four months. On examination the abdomen appeared a little swollen and there was tenderness in the right lower quadrant. There was resistance to palpation and slight dullness on percussion. On August 18, 1958, a laparotomy was performed. The surgeon, who had not operated on the elder brother was aware of the family history and he read the case notes and post mortem report on L.B before the operation. He reported very similar findings—a large, hard, irregular. mass which appeared to arise in the hepatic flexure and spread to invade the psoas muscle, right kidney, mesenteric and para-aortic nodes. Two mesenteric lymph nodes were examined histologically and one of these showed the structure of a reticulum cell sarcoma (Fig. 5 and 6). This patient was given small doses of deep X-ray therapy receiving 1,500 r. in all. He did improve a little and the mass appeared to get smaller. On November 6, 1958, an examination was made under general anaesthesia and the tumour was found to occupy the right half of the abdomen from the right costal margin to the iliac fossa.—This patient belonged to blood group AB and was rhesus negative. The white blood count was not recorded higher than 10,000 per cu. mm. with 91 per cent neutrophils. The haemoglobin fell as low as 6·9 g. He lived about three months after the diagnosis was established and continued to lose weight and died on November 13, 1958.

*Post mortem findings.*—The autopsy was performed 18 hours after death and a full examination was carried out. There was a thrombus attached to the wall of the lower part of the inferior vena cava and partially occluding it. Peritonitis was present and this had been caused through a perforation of the small bowel near the ileo-caecal region. The main features were in the alimentary tract. Fibrinous exudate covered a large part of the small and large intestine. There were many adhesions of the small bowel on itself and to the parietal peritoneum. Most of these could be broken down with pressure but some around the right iliac fossa were very firm. The terminal ileum, caecum and lower part of the ascending colon showed a thickened wall and the appearances were those of infiltration with tumour (Fig. 7). The mucosa was intact but stretched. The ileo-caecal valve was thickened. The vermiform appendix was caught up in some adhesions and appeared to be replaced by tumour. The remainder of the small and large intestines appeared normal. The liver contained a large deposit of tumour measuring 3 cm. across. This was in the lower part of the right lobe and the hepatic flexure was adherent here. There were enlarged lymph nodes in the mesentery, at the ileo-caecal region and around the coeliac axis. The spleen was small, soft and normal, in appearance. The kidneys showed no abnormality.

*Histological examination.*—The tumour in the small and large intestines, lymph nodes, vermiform appendix (Fig. 8) and liver had a uniform appearance. There were masses of polyhedral cells, fairly even in size with scanty cytoplasm and prominent hyperchromatic nuclei. Some of the nuclei were crenated and others were pyknotic. Mitotic figures occurred. Much of the tumour was degenerate and in parts there was hyaline material (Fig. 9) and broad bands of collagen. Reticulin preparations showed reticulin fibres surrounding tumour cells. The appearances were those of a reticulum cell sarcoma.

### DISCUSSION

Koller (1959) points out that certain types of tumour show a high susceptibility to inheritance. Amongst these tumours are retinoblastoma, neurofibromastosis, multiple polyposis and xeroderma pigmentosum. In the first three types of tumour the disease is transmitted by a dominant gene, while in the last the gene is recessive. Brief review of studies of cancer incidence in monozygotic twins show that the presence of predisposition does not decide the fate of an individual, it is the outcome of the environmental influence acting upon the genetic constitution.

Devore and Doan (1957) in a familial study of 440 carefully verified cases of Hodgkin's disease found fifteen families in which proven multiple cases of Hodgkin's disease or other lymphomata developed in blood relatives with or without direct contact and in some instances with an interval of some years between.

McConnell (1958) found a nasopharyngeal carcinoma (confirmed by lymph node biopsy) in a female child aged 8 years and her brother developed a similar tumour at the age of 8 years. Both children died of secondaries in the liver some months after diagnosis.

Howel-Evans *et al.* (1958) described an association between keratosis palmaris et plantaris (tylosis) and carcinoma of the oesophagus in two Liverpool families. Eighteen cases of carcinoma of the oesophagus have occurred. There is unequivocal evidence that the neoplasm was associated with tylosis in seventeen patients. No case of oesophageal carcinoma has occurred in members unaffected with tylosis. Tylosis is inherited as an autosomal dominant.

Herrell, Ruff and Bayrd (1958) record the occurrence of multiple myeloma in two siblings aged 53 and 55 years when first seen.

Regarding the nature of the tumour Mestel (1959) reports thirteen cases of lymphosarcoma in the young (including one of reticulum cell sarcoma) considered to be confined to the small intestine at time of diagnosis. Twenty five further cases are found in the literature. The disease is most common in the 3–8 year old group and five and a half times more prevalent in the male than the female. The prognosis is bad, the average length of survival being just over thirteen months.

Rosenberg *et al.* (1958) commenting on lymphosarcoma (which includes giant follicular lymphosarcoma and reticulum cell sarcoma) from the records of thirty years at the Memorial Centre of New York state that amongst 1269 cases of lymphosarcoma 69 cases were 15 years of age and below. In the older children males show predominance but in the very young the ratio approaches unity. The disease in children presents with abdominal symptoms about twice as often as in adults and the bowel is often involved. In 11 cases in this series the disease was fatal as a result of rapid uncontrolled local disease before spread occurred. This picture was somewhat more common in reticulum cell sarcoma. Of 26 children on whom autopsies had been performed about three quarters had involvement of the retroperitoneal lymph nodes and just over half had foci in the small intestine. Half the children were dead in seven months. The disease seems more aggressive in childhood. Certain sites, other than in lymph nodes, appear to be the primary focus more frequently in children than in adults.

In this particular family the parents of the siblings described were anxious to ascertain whether they could have further offspring with impunity and at present have decided against it. Dr. Fraser Roberts (1958, personal communication) was consulted and he wrote that he considered it unlikely that the condition is due to a chance coincidence. A single gene can sometimes do what is usually determined by a non-genetic error of development. There is difficulty in giving any genetic prognosis but the risk of recurrence on a further child is probably appreciable. The worst it could be would be one in four (due to a recessive gene) but the genetics might be more complicated and the more complex the genetics the better the outlook in terms of odds.

### SUMMARY

The cases of two brothers who died in the third year of life from a reticulum cell sarcoma are described. The diagnosis was established during life, in each case by biopsy and in the second case by exploratory laparotomy.

Full autopsies were carried out on both cases, the tumour affecting the terminal ileum, caecum, ascending colon and lymph nodes.

The heredity nature of tumours is briefly discussed. The question arises as to what advice can be given to the parents as to the possibility of any further offspring being similarly affected.

Malignant lymphomata in children are briefly considered.

### REFERENCES

Devore, J. W. and Doan, C. A.—(1957) *Ann. intern. Med.*, **47**, 300.

Herrell, W. E., Ruff, J. D. and Bayrd, E. D.—(1958) *J. Amer. med. Ass.*, **167**, 1485.

Howel-Evans, W., McConnell, R. B., Clarke, C. A. and Sheppherd, P. M.—(1958) *Quart. J. Med.*, **27**, 413.

Koller, P. C.—(1959) *Practitioner*, **182**, 693.

McConnell, E. M.—(1958) *Brit. J. Cancer*, **12**, 195.

Mestel, A. L.—(1959) *Ann. Surg.*, **149**, 87.

Rosenberg, S. A., Diamond, H. D., Dargeon, H. W. and Craver, L. F.—(1958) *New Engl. J. Med.*, **259**, 505.